# ISR8/IRF1-AS1 Is Relevant for IFNα and NF-κB Responses

**DOI:** 10.3389/fimmu.2022.829335

**Published:** 2022-07-04

**Authors:** Marina Barriocanal, Laura Prats-Mari, Nerea Razquin, Celia Prior, Juan Pablo Unfried, Puri Fortes

**Affiliations:** ^1^Department of Gene Therapy and Regulation of Gene Expression, Center for Applied Medical Research (CIMA), University of Navarra (UNAV), Pamplona, Spain; ^2^Navarra Institute for Health Research (IdiSNA), Pamplona, Spain; ^3^Liver and Digestive Diseases Networking Biomedical Research Centre (CIBERehd), Madrid, Spain; ^4^Spanish Network for Advanced Therapies (TERAV ISCIII), Madrid, Spain

**Keywords:** IFNα, NF-κB, IRF1, ISGs, XIST, ZNF, inflammation, autoimmune diseases

## Abstract

The study of the interferon (IFN) α-induced cell transcriptome has shown altered expression of several long non-coding RNAs (lncRNAs). *ISR8/IRF1-AS1* (IFN stimulated RNA 8), located close to IFN regulatory factor 1 (*IRF1*) coding gene, transcribes a lncRNA induced at early times after IFNα treatment or IRF1 or NF-κB activation. Depletion or overexpression of ISR8 RNA does not lead to detected deregulation of the IFN response. Surprisingly, disruption of *ISR8* locus with CRISPR-Cas9 genome editing results in cells that fail to induce several key ISGs and pro-inflammatory cytokines after a trigger with IFNα or overexpression of IRF1 or the NF-κB subunit RELA. This suggests that the *ISR8* locus may play a relevant role in IFNα and NF-κB pathways. Interestingly, IFNα, IRFs and NF-κB-responding luciferase reporters are normally induced in ISR8-disrupted cells when expressed from a plasmid but not when integrated into the genome. Therefore, IFNα and NF-κB pathways are functional to induce the expression of exogenous episomic transcripts but fail to activate transcription from genomic promoters. Transcription from these promoters is not restored with silencing inhibitors, by decreasing the levels of several negative regulators or by overexpression of inducers. Transcriptome analyses indicate that ISR8-disrupted cells have a drastic increase in the levels of negative regulators such as XIST and Zinc finger proteins. Our results agree with *ISR8* loci being an enhancer region that is fundamental for proper antiviral and proinflammatory responses. These results are relevant because several SNPs located in the *ISR8* region are associated with chronic inflammatory and autoimmune diseases including Crohn’s disease, inflammatory bowel disease, ulcerative colitis or asthma.

## Introduction

The interferon (IFN) pathway is a key cellular mechanism to counteract infections (1). Bacteria and viruses produce pathogen-associated molecular patterns (PAMPs) recognized by intracellular or extracellular PAMP recognition receptors (PRRs). Upon stimuli, these PRRs activate several pathways leading to activation of IFN response factors (IRF3 and IRF7) and NF-κB (nuclear factor kappa-light-chain-enhancer of activated B cells) transcription factors, which induce the expression of proinflammatory cytokines (CXCL10, IL6, IL12) and type I IFN (IFNα and IFNβ) (2). Auto- and paracrine recognition of the released IFN activates the JAK/STAT pathway. Then, signal transducer and activator of transcription (STAT1 and STAT2) proteins are phosphorylated, form heterodimers and bind IRF9 to form the IFN stimulated gene factor 3 (ISGF3) complex. ISGF3 translocates to the nucleus and binds to the IFN-sensitive response element (ISRE) promoter and enhancer regions that contact each other. Enhancer-promoter interactions allow the correct spatial architecture of the chromosome, including topologically associated domains (TADs) flanked by insulator elements as the CCCTC-binding factor (CTCF) (3, 4). The exact mechanism of enhancer-promoter binding is not fully understood; however it is clear that Mediator and Cohesin proteins play important roles. Cohesin connects two DNA segments by forming rings, and Mediator connects with cohesin to stabilize enhancer-promoter interactions (5, 6). Then, Mediator helps RNA polymerase (pol) II loading to the promoters and pre-initiation complex (PIC) formation. In some cases transcription starts, but RNA pol II is paused by the negative elongation factor (NELF) and the DRB-sensitivity inducing factor (DSIF). BRD4 binding to enhancers recruits CDK9 and allows the positive transcription elongation factor b (pTEFb) to phosphorylate NELF and DSIF. Phosphorylated NELF is evicted from the transcription complex, whereas DSIF becomes a positive factor, thus allowing elongation and gene transcription (7–9). After ISGF3 translocation, RNA pol II elongation induces hundreds of IFN stimulated genes (ISGs). Most of them are positive regulators that will act to clear the infection (IRF1, GBP1) while others help the cell return to homeostasis (USP18 or SOCS) (8, 10, 11). While most ISGs described to date transcribe for proteins, the type I IFN pathway can also induce the expression of short and long non-coding RNA (lncRNA) ISGs (12).

LncRNAs are non-coding transcripts longer than 200 nucleotides with poor coding potential. Compared to mRNAs, lncRNAs are less conserved and they tend to be more tissue specific, and to localize preferentially to the nucleus (13–17). Similarly to mRNAs, most lncRNAs are transcribed from RNA pol II and may be spliced and polyadenylated. Although most lncRNAs remain unstudied, it has been clearly shown that some lncRNAs have important regulatory functions that can be performed in *cis* or *trans*. Enhancer RNAs (eRNAs) are a subset of *cis*-acting lncRNAs transcribed from enhancer regions. Although eRNAs were thought to be byproducts of enhancer transcription, some eRNAs are required for enhancer function and transcriptional activation of coding genes located in the same territory (18, 19). In fact, some eRNAs can reinforce DNA looping, including enhancer-promoter interaction and stability, and mRNA transcription by affecting RNA pol II progression and TF recruitment to the promoter region (20, 21). *Arc* eRNA for example interacts with NELF helping its release (20). Interestingly, some lncRNAs function in a sequence-independent manner as it is the mere act of transcription what drives their mechanism of action (22).

The number of lncRNAs described to play a role in the immune response is increasing (12). Several lncRNAs regulate the expression of ISGs or proinflammatory genes positively or negatively. LncRNA Cox2 is a lipopolysaccharide (LPS)-induced lncRNA that regulates ISG expression by interacting with heterogeneous ribonucleoproteins (hnRNP) -A/B and -2A/B (23). The negative regulator of IFN response (NRIR) is induced by IFN, blocks ISG transcription and favors viral replication (24). Eosinophil granule ontogeny transcript (EGOT) is induced by NF-κB, viral infection and other stress signals and modulates ISG levels and NF-κB targets by affecting the NF-κB transcription coactivator TBLR1 (25, 26).

Previously, we have identified several lncRNAs whose expression is induced by IFNα treatment of HuH7 cells (27, 28). One of them is the IFNα-stimulated RNA 8 (ISR8)/IRF1-AS1, transcribed from a gene located tail-to-tail to *IRF1*. In this work, we show that disruption of *ISR8* using the CRISPR-Cas system results in clones with abrogated IFNα and NF-κB signaling. Interestingly, depletion of ISR8 RNA does not affect IFNα signaling, and overexpression of ISR8 RNA does not recover the IFNα response in *ISR8*-disrupeted cells, suggesting that *ISR8* locus play an essential regulatory role at DNA level that can be tracked by ISR8 RNA expression.

## Material and Methods

### Cells and Cell Culture

HeLa cells derived from epithelial cervix cancer were obtained from ATCC. HuH7 hepatocarcinoma cells were provided by Dr. Chisari’s laboratory (Scripps Research Institute, La Jolla, CA, USA). Hap1 were obtained from Horizon Discovery and were grown in Iscove´s Modified Dulbecco’s Medium (IMDM). HeLa and HuH7 were maintained in Dulbecco’s Modified Eagle Medium (DMEM). Media were enriched with 10% fetal bovine serum (FBS) and 1% penicillin-streptomycin, and incubated in a 5% CO_2_ atmosphere. Twenty-four hours before transfection or treatment, cells were seeded in 6-well plates. After approval from the Ethics and Scientific Committees, peripheral blood mononuclear cells (PBMCs) and macrophages were kindly isolated from healthy donors by Dr. Sandra Hervás (CIMA, University of Navarra, Spain) and used immediately after their isolation. These cells were maintained in RPMI media enriched with 10% fetal bovine serum (FBS) and 1% penicillin-streptomycin, and incubated in a 5% CO_2_ atmosphere. For macrophage isolation, monocytes were sorted from human PBMCs with anti-CD14 microbeads (Miltenyi). Then 0,5 x 10^6^ cells/ml were cultured in enriched medium supplemented with 10 ng/ml M-CSF (Immunotool) for up to 7 days. Macrophages were CD14^+^ and CD80^-/low^ as evaluated by FACS.

### Plasmids

For ISR8 overexpression, cDNA from HeLa cells was amplified with the primers described in **Supplementary Table 1** with Phusion High-Fidelity DNA polymerase (#F530S, Thermo Scientific). The amplified product was cloned into a pGEM-T Easy intermediate vector (#A1360, Promega) and the resulting plasmid (pGEM-TISR8) was verified by sequencing. Then, pGEM-TISR8 was digested with EcoRI and ClaI and was cloned into the same sites of pCAGGS (#LMBP 2453, BCCM) to obtain the mammalian expressing plasmid pCAGGS-ISR8. pCMV6-XL5-empty and pCMV6-XL5-IRF1 plasmids were kindly provided by Dr. Larrea (CIMA, University of Navarra, Spain) (29). IRF3 expressing pIRF3 plasmid was obtained from Dr. Nistal-Villán (University San Pablo, CEU) (30). pRELA plasmid expressing p65/RELA was obtained with the mediation of addgene (#21984, Addgene) (31). Plasmid containing p300 acetylase was obtained from Dr. Revilla (CBM/CSIC, Madrid) (32). Luciferase reporter gene responding to: NF-κB (pNF-κB-Luc) was purchased from Clontech (NF-κB 3xLuc), to IFN (pISRE-Luc) was obtained from Dr. Nistal-Villán (University San Pablo, CEU) (33) or to IRF (pIRF-Luc, (PRDIII-I)4-Luc) was obtained from Dr. Ludwig (Universitat Munster) (34). The plasmid pIRF·ISRE-Luc was obtained from Dr. Hernández-Alcoceba (CIMA, University of Navarra, Spain) (33). Promoter and reporter sequences from pIRF·ISRE-Luc were obtained by digestion with EcoRI and XbaI (New England BioLabs), and were inserted into the same sites of the sleeping beauty pSB-Puro (Addgene #60523) modified to express cherry (Rovira et al., in preparation). The resulting pSBIRF·ISRE-Luc vector was co-transfected with the SB transposase expression vector (Addgene #127909) to generate stable cells. The plasmid expressing luciferase reporter from a CMV promoter (pCMV-Luc) was used as a negative control (35) and the plasmid expressing a Renilla reporter gene (pCMV-RL, Promega) was used in all cases as a transfection and loading control.

CRISPR-Cas9 gene-editing system was used to disrupt ISR8 locus following the protocol established by Zhang’s lab (36). Primers with guide sequences to disrupt ISR8 promoter are described in **Supplementary Table 1**. Primers were hybridized and cloned into pX334-U6-DR-BB-DR-Cbh-NLS-hSpCas9n(D10A)-NLS-H1-shorttracr-PGK-puro and pX335-U6-Chimeric_BB-CBh-hSpCas9n(D10A) plasmids (#42333 and #42335 respectively, Addgene) (37). In order to obtain homologous recombination, the guide-expressing plasmids were transfected into HeLa or Hap 1 cells together with a DNA fragment containing a neomycin or a puromycin resistance gene without promoter flanked by sequences homologous to those surrounding each ISR8 guide RNA target. These cassettes were amplified by PCR (Phusion High-Fidelity DNA polymerase #F530S, Thermo Scientific) from a pGemT plasmid (#A3600, Promega) containing the neomycin or puromycin resistance genes followed by polyadenylation sequences. The PCR reaction was performed with primers described in **Supplementary Table 1**. Twenty-four hours after transfection positive clones were selected in media containing 1250 µg/ml (for HeLa) or 1400 µg/ml (for Hap1) of neomycin (Gibco) or 1 µg/ml of puromycin (Gibco) for 3 weeks, single clones were amplified and validated by PCR with the primers described in **Supplementary Table 1**.

### Virus Infection

Encephalomyocarditis virus (EMCV) was kindly provided by Dr. Esther Larrea (CIMA, University of Navarra, Spain) and amplified in HeLa cells. Cells were plated in a 150 cm^3^ flask, infected with 100 µl of an EMCV stock, diluted in 5 ml of DMEM and incubated at 37°C for 1h. Then, 15 ml of media were added and infection was allowed to proceed for 24h at 37°C. Finally, the supernatant was collected, the detritus eliminated by centrifugation, and the virus was titrated. To this aim, HeLa cells and pNISR8 clones were seeded into 96-well plates with 100 µl of DMEM. One day later, 100 µl of fresh media were added with 1:5 serial dilutions of the EMCV stock. Cell death was detected under a microscope 17h later. Then, the cells were washed 2 times with phosphate-buffered saline (PBS) and stained with crystal violet for 10 min in a platform rocker shaker. The crystal violet was washed extensively in PBS and cells were lysed with 100 µl of SDS 0.1% for 4h. Absorbance was measured at λ=540 in the Multiskan Ascent equipment (Mtx lab systems) and analyzed with the Ascent Software [modified from (38)]. According to our results, a 1:125 dilution of EMCV stock was used to evaluate the antiviral effect of IFN in HeLa cells and pNISR8 clones.

### Transfections and Treatments

Plasmid (1 µg), gapmer (0.05 nmol) (Exiqon) and siRNAs (0.08 nmol) (Sigma-Aldrich) transfections were performed into 6 well plates using Lipofectamine 2000 (#11668019, Invitrogen) according to manufacturer’s recommendations. The sequence of the siRNAs and gapmers used is listed in **Supplementary Table 1**. LPS (DIFCO) kindly provided by Dr. Lasarte (CIMA, University of Navarra, Spain) was used at a final concentration of 5 µg/ml together with 15 µg/ml polyinosinic-polycytidylic acid [poly (I:C)] (*In vivo*gen). Tumor necrosis factor alpha (TNFα) (#300-01A, Prepotech) was used at a final concentration of 20 ng/µl. Tunicamycin (#T7765-5MG, Sigma-Aldrich) was kindly provided by Dr. Aragón (CIMA, University of Navarra, Spain) and was used at a final concentration of 1 µg/ml. Treatments with JQ1 and I-BET151 (GSK1210151A) bromodomain inhibitors (#SML0974-5MG and #SML0666-5MG, Sigma-Aldrich) were performed at 1 or 10 µM, and 250 or 1000 nM respectively. Treatment with flavopiridol (#52679, Selleckchem) was performed at 100 or 300 nM. Treatment with Panobinostat (LBJ589; #HY-10224) was performed at 50 μM and Azacytidine (#HY-111644) at 3000 μM. The inhibitors GSK126 (#HY-13470), UNC1999 (#HY-15646), CM272-G9a (HY-101925) and CCT251545 (#HY-12681) were used at a concentration of 30 μM, 15 μM, 150 μM and 80 nM, respectively.

When indicated cells were treated with 1000 U/ml human-IFNα5b (Lot: 060505-03T, Sicor Biotech) for the designated times. For EMCV experiments 11 U/ml of IFNα followed by 1:3 serial dilutions were added to the cells 24h prior to infection. Cells were seeded in M6 well plates 24 h previous to any transfection or treatment, except in EMCV, that 96 well plates were used.

### Western Blot and Luciferase Measurement

For western blot analysis, we used GAPDH (Sigma), IRF1 (sc-497, Santa Cruz), NF-κB p65 (#8242, Cell Signaling) and NF-κB p65 acetyl K310 (ab19870, AbCam) antibodies. Thirty µg of protein in RIPA buffer were denatured at 95°C for 5 min, and run through a 12% polyacrylamide gel and transferred onto a nitrocellulose membrane (Protran Whatman) (39). After transfer, membranes were blocked in 5% milk/TBST for 1h and incubated with monoclonal antibodies against GAPDH diluted 1:10000, or IRF1, NF-κB p65 or NF-κB p65 acetyl K310 diluted 1:1000. After washing, secondary anti-mouse antibody conjugated with peroxidase diluted 1:10000 (Sigma) and anti-rabbit antibody conjugated with peroxidase diluted 1:5000 (Cell Signalling) were used. Western blots were developed with ECL (Perkin-Elmer). Renilla and firefly luciferase activities were measured using the Dual Luciferase System (Promega) in a Berthold Luminometer (Lumat LB 9507) as previously described (40). The values obtained for firefly luciferase were corrected for equal transfection efficiency with Renilla luciferase activity.

### RNA Extraction, Sequencing, RT and Quantitative PCR

RNA extraction from treated cells was performed using the MaxWell 16 research system from Promega, following the manufacturer’s recommendations. The RNA concentration was measured using a NanoDrop 1000 Spectrophotometer. For high-throughput sequencing, RNA of excellent quality (n=1), as measured by TapeStation (Agilent), was sent to Macrogen, where total RNA was sequenced with Illumina TRuSeq stranded mRNA kit. Sequences were paired-end, 150 bases long and strand-specific. For reverse transcription, 1 µg of RNA was incubated in M-MLV-RT buffer, 5 µM DTT, 200 units M-MLV-RT enzyme (#28025013, Invitrogen), 0.5 mM dNTPs (#10297018, Invitrogen) and 10 ng/ml random primers (#48190011, Invitrogen) in a final volume of 40 µl. The reaction was set at 37°C for 60 min and 95°C for 1 min in the C1000 Touch Thermal Cycler from Bio-Rad and immediately placed at 4°C. Quantitative polymerase chain reaction was performed with 5 µl Syber-Green mix (#1708880, Bio-Rad), 0.27 µM of each primer and 2µl of the cDNA mix in a final volume of 11 µl in the CFX96 Real-Time system from Bio-Rad. The mixture was incubated at 95°C for 3 min, then at 95°C for 15 s, 60°C for 15 s and 72°C for 25 s for 34 cycles, and finally, 1 min at 95°C and 1 min at 65°C. The results were analyzed with Bio-RadCFX manager software. The primers used were designed using the Primer3 program (http://bioinfo.ut.ee/primer3-0.4.0/primer3/) and are listed in **Supplementary Table 1**. Relative expression (rel exp) was calculated as 2 to the power of the minus delta of the cycle threshold (Ct) of the gene evaluated and the Ct of the *GAPDH* housekeeping.

### Formaldehyde-Assisted Isolation of Regulatory Elements and Chromatin Immunoprecipitation

FAIRE was performed according to (41). In brief, 1 x 10^7^ HeLa or pNISR8 cells were crosslinked for 5 minutes with 37% formaldehyde added directly in the media to a final concentration of 1%. Then, cells were lysed by bead beating and extracts were sonicated for increasing cycles of 30 seconds in a Bioruptor Standard (Diagenode #UCD-200). A portion of the sample was used to evaluate DNA fragmentation by electrophoresis in agarose gels after crosslinking reversion with proteinase K (Thermo Scientific #EO0491). When DNA fragments ranged between 150-750 bps, the crosslinked fractions were treated with phenol/chloroform to extract accessible DNA and the levels in ISR8 region were evaluated by qPCR using a reference sequence as normalizer. Primers used are described in **Supplementary Table 1**.

For CHIP assay, cells were crosslinked with 37% formaldehyde at a final concentration of 1%. Cell lysis was performed in lysis buffer containing 0.5% NP-40 (Sigma-Aldrich, #85124) and using a 2mL Dounce homogenizer pestle B (Kimble, #885303-0002). Cell lysate was sonicated (Bioruptor Standard) for 5 cycles of 30s and centrifuged at 19000G for 10 minutes to discard insoluble nuclear fraction. Soluble fraction was incubated with magnetic beads coated with RELA K310 (#ab19870) and Ser2 phosphorylated polymerase II (#ab193468) antibodies or control IgG. Samples were eluted, and crosslinking was reversed with proteinase K (Thermo Scientific #EO0491). Purification of DNA by phenol/chloroform extraction was carried out and DNA was analyzed by qPCR. DNA obtained from input samples was also analyzed as a reference. Primers used are described in **Supplementary Table 1**.

### Statistics and Bioinformatic Analyses

RNA sequencing data analysis was performed as described (28). HiC data was extracted with HiGlass and enrichment was evaluated with EnrichR. Statistical analysis was performed using GraphPad. Statistical significance was calculated using a two-tailed nonparametric Mann–Whitney U-test for samples that do not follow a normal distribution. When the samples followed a normal distribution according to the Shapiro–Wilk test, a two-tailed Student’s t-test was used. P-values lower than 0.05 were deemed as significant. In all data shown, * denotes P ≤ 0.05, **P ≤ 0.01, ***P ≤ 0.001, and ****P ≤ 0.0001 while ns indicates non-significant differences.

## Results

### ISR8 Transcript Is Induced by IFNα in Several Cells

**Figure 1:**
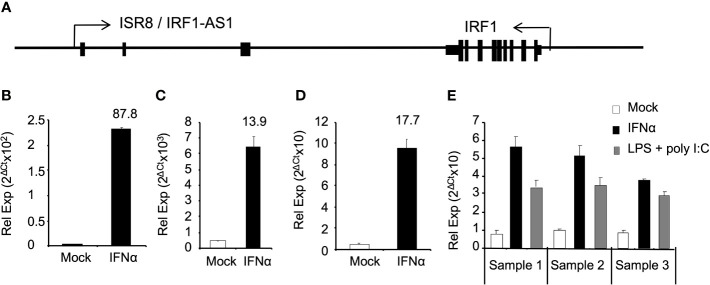
ISR8/IRF1-AS1 is induced by IFNα in different cell lines. **(A)** Schematic of *ISR8/IRF1-AS1* and *IRF1* coding gene. **(B–E)**. HUH7 **(B)** and HeLa **(C)** cells, macrophages **(D)** and PBMCs **(E)** were treated with none or 1000 U/ml IFNα for 6h. PBMCs were also treated with 5 µg/ml LPS plus 15 µg/µl poly (I:C) for 6 h **(E)**. *ISR8* expression levels were measured by RT-qPCR. GAPDH mRNA was also evaluated and used as a reference. Error bars indicate standard deviations. Fold increase is indicated at the top of each bar. Experiments were performed at least twice and a representative figure is shown.

We have previously shown that *ISR8/IRF1-AS1* is located tail-to-tail to the *IRF1* gene and transcribes for an IFNα-induced lncRNA (**Figure 1A**) (27). To determine whether *ISR8* is also induced by IFNα in primary cells and cell lines from different origin, HuH7, HeLa, purified macrophages and PBMCs obtained from three healthy donors were incubated with IFNα for 6h and ISR8 lncRNA was evaluated by RT-qPCR. The results indicate that IFNα treatment induces the levels of ISR8 up to 87-fold in these cells (**Figures 1B–E**). Similar results have been observed with A549, HEK293 or THP1 cells, while purified CD4 cells did not induce ISR8 after IFNα treatment for 6h (27). Treatment of PBMCs with IFN-inducing agents such as LPS and poly (I:C) also increased ISR8 expression (**Figure 1E**).

### IFNα-Mediated Induction of Several ISGs Is Abrogated in *ISR8*-Disrupted Cells

**Figure 2:**
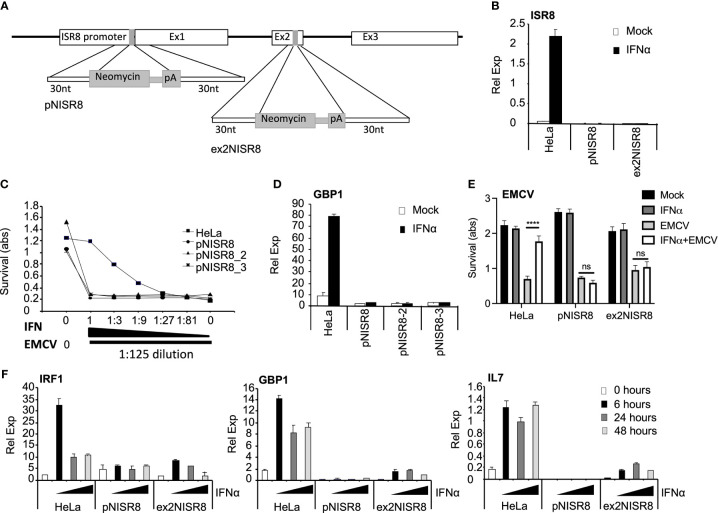
Analysis of *ISR8*-disrupted clones obtained by CRISPR-Cas9 genome editing technique. **(A)** Schematic of the neomycin cassette and the insertion in *ISR8* promoter and exon 2 to obtain the pNISR8 and ex2NISR8 stable cell lines. **(B)** ISR8 mRNA levels in the indicated cells treated with 0 or 1000 U/ml IFNα for 6 h. **(C)** Survival of the indicated cells after treatment for 6h with decreasing doses of IFNα (0, 0.045, 0.123, 0.41, 1.23, 3.7 and 11.11 U/ml IFNα) and EMCV infection for 18h. **(D)** GBP1 mRNA levels in the indicated cells treated with 0 or 1000 U/ml IFNα for 6 h. **(E)** Similar to C but with 11.11 U/ml IFNα. **(F)** GBP1, IRF1 and IL7 mRNA levels were measured in the indicated cells treated with 0 or 1000 U/ml IFNα for the indicated times. GAPDH mRNA was also measured in all RT-qPCR evaluations and used as a reference. Error bars indicate standard deviations. Experiments were performed at least four times except C and E, which were performed twice and a representative figure is shown.

To determine whether ISR8 plays a role in the IFN response, we altered the *ISR8* locus using CRISPR-Cas9 genome editing (**Figure 2A**). First, we targeted the *ISR8* promoter by introducing a cassette containing a neomycin (neo) resistance gene without promoter followed by polyadenylation sequences. We determined that the selected neo resistant clones had introduced the neo cassette in the *ISR8* locus and that they did not express ISR8 (**Figure 2B** and **Supplementary Figure 1A**). To determine whether these clones, called pNISR8 (from ISR8 promoter::NeopA), have a proper antiviral response, we performed a survival assay to a lethal amount of encephalomyocarditis virus (EMCV) with increasing doses of IFNα (see material and methods for details). Survival was evaluated in HeLa cells and clones pNISR8, pNISR8_2 and pNISR8_3. As expected, all IFNα-untreated cells died after infection, while non-infected cells showed similar survival rates (**Figure 2C**). Surprisingly, while infected HeLa cells showed higher survival with increasing IFNα, infected pNISR8 cells were insensitive to the antiviral effect of IFNα. In agreement with this, IFNα treatment for 6h failed to induce the expression of ISGs such as *GBP1* or *IL7* in the pNISR8 clones compared to control HeLa cells (**Figure 2D** and **Supplementary Figure 2A**).

To confirm these results, we generated new ISR8-disrupted cells by introducing the neo cassette at the second exon of ISR8 (**Figure 2A**). The ISR8-disrupted neo-resistant clone was called ex2NISR8 (from ISR8exon2::NeopA) (**Figure 2B** and **Supplementary Figure 1B**). This clone also failed to survive EMCV infection in the presence of IFNα (**Figure 2E**). To evaluate ISG induction in response to IFNα, ISG mRNA levels were quantified in HeLa, pNISR8 and ex2NISR8 cells treated with IFNα for 0, 6, 24 or 48h (**Figure 2F**). While IRF1, GBP1 and IL7 mRNAs were induced by IFNα in HeLa cells, IFNα failed to induce the expression of these ISGs in pNISR8 cells and led to milder induction in ex2NISR8 cells. Finally, to determine whether this effect could also be observed in another cell line, we introduced the neo or a puromycin (pur) resistance gene followed by polyadenylation sequences after the *ISR8* promoter of Hap 1 cells. Then, two antibiotic resistant clones of each type were expanded and named Hap1pNISR8 (with NeopA insertion) and Hap1pPISR8 (with PurpA insertion). Similar to what was observed before, IFNα treatment for 6h failed to induce the expression of ISGs such as *GBP1* in the pNISR8/pPISR8 clones compared to control Hap1 cells (**Supplementary Figure 2B**).

### Inhibition or Re-Expression of ISR8 Does Not Affect ISG Induction by IFNα

**Figure 3:**
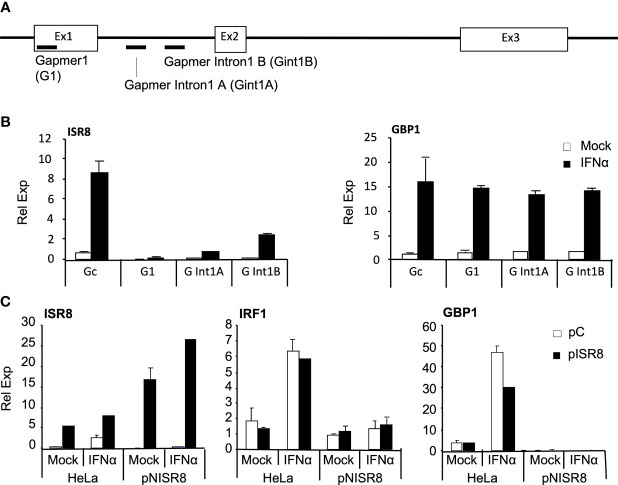
Analysis of ISG induction after transient inhibition or overexpression of ISR8. **(A)** Schematic of gapmer binding to ISR8 sequences. **(B)** ISR8 and GBP1 mRNA levels in HeLa cells transfected with control (Gc) or ISR8 gapmers for 48 h and treated with 0 or 1000 U/ml IFNα for 6h. **(C)** ISR8, IRF1 and GBP1 mRNA levels in the indicated cells transfected with a control plasmid (pC) or pCAGGS-ISR8 (pISR8) for 48 h and treated with 0 or 1000 U/ml IFNα for 6h. GAPDH mRNA was also evaluated and used as a reference. Error bars indicate standard deviations. Experiments were performed at least four times and a representative figure is shown.

The effects described so far could result from a genomic alteration at the *ISR8* locus, deficient transcription, or the depletion of ISR8 transcripts. To discriminate between these possibilities, we evaluated the effect of exogenous ISR8 expression and ISR8 inhibition with gapmers. HeLa cells were transfected with gapmers targeting ISR8 lncRNA (G1) or ISR8 pre-lncRNA at intron 1 (Gint1A and B) (**Figure 3A**). Two days later, cells were treated with IFNα for 6h and the RNA levels of ISR8 and GBP1 were evaluated by RT-qPCR. All gapmers decreased ISR8 levels efficiently (**Figure 3B**). However, ISR8 depletion did not affect the mRNA levels of GBP1 or other ISG transcripts evaluated (**Figure 3B** and data not shown).

For ISR8 re-expression, enough pCAGGS-ISR8 was transfected into pNISR8 cells to obtain ISR8 levels similar to those observed in IFNα-induced HeLa cells. HeLa cells transfected with the same amount of pCAGGS-ISR8 and cells transfected with an empty pCAGGS were used as controls. Two days later, cells were mock-treated or treated with IFNα for 6h and RNA levels of ISR8, IRF1 and GBP1 were evaluated (**Figure 3C**). The results show that IRF1 and GBP1 mRNA levels are not affected by ISR8 expression in HeLa or pNISR8 cells. Similar results were observed when ISR8 was re-expressed in ex2NISR8 or in Hap1pNISR8 cells (**Supplementary Figure 2C**). As a whole, these results indicate that the ISR8 transcript does not cause the defect observed in *ISR8*-disrupted cells.

### Defective ISG Induction by IFNα in *ISR8*-Disrupted Cells Is not Due to IRF1 Deficiency or an Altered NF-κB Pathway

**Figure 4:**
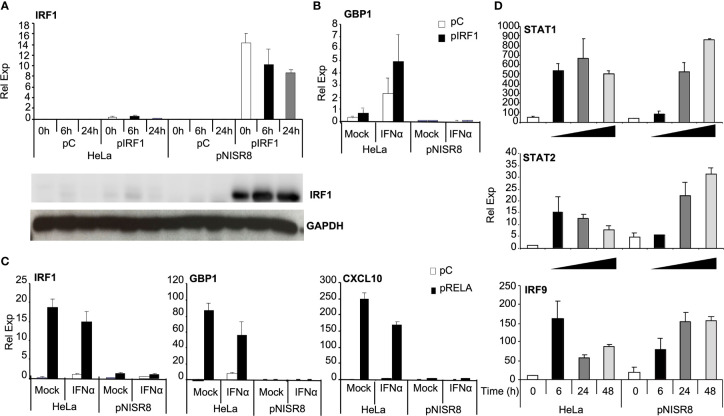
Analysis of the induction of ISGs and proinflammatory genes in *ISR8*-disrupted cells after IRF1 or RELA overexpression and IFNα treatment. **(A)** IRF1 mRNA was evaluated by RT-qPCR and IRF1 was visualized by Western-blot using GAPDH as a reference in the indicated cells transfected with a control plasmid (pC) or pIRF1 and treated with 0 or 1000 U/ml IFNα for the indicated times. **(B)** GBP1 mRNA levels were evaluated in cells treated as in A but IFNα was evaluated only for 6h. **(C)** IRF1, GBP1 and CXCL10 mRNAs were evaluated in cells treated like in B but transfected with pRELA. **(D)** STAT1, STAT2 and IRF9 mRNAs in the indicated cells treated with 0 or 1000 U/ml IFNα for the indicated times. GAPDH mRNA was used as a reference. Error bars indicate standard deviations. Experiments were performed at least four times and a representative figure is shown.

*ISR8*-disrupted clones fail to induce several ISGs, including *IRF1* (**Figure 2F**). *IRF1* expresses a transcription factor that binds the promoter of several ISGs to induce their expression after IFNα treatment (42, 43). The promoter regions of *GBP1* and *IL7* have target sites for IRF1, according to ChIP experiments performed by ENCODE (44). As *IRF1* is located close to the *ISR8* locus (**Figure 1A**), we hypothesized that *ISR8*-disruption would affect *IRF1* induction by IFNα, resulting in a defective activation of several ISGs (**Figure 2F**). To determine whether IRF1 re-expression could recover ISG induction in pNISR8 cells, we transfected HeLa and pNISR8 cells with a control or an IRF1-expression plasmid (pCMV6-XL5-IRF1, called pIRF1 for simplicity). Forty-eight hours later, we treated the cells with IFNα for the indicated times. Then, extracts were collected, and we evaluated IRF1 expression by Western-blot and the mRNA levels of several ISGs by RT-qPCR. Surprisingly, IRF1 expression was very high in pIRF1-transfected pNISR8 cells compared to HeLa cells (**Figure 4A**). As expected, overexpression of IRF1 resulted in a significant increase of GBP1 mRNA levels in control cells or IFNα-treated HeLa cells (**Figure 4B**). Instead, GBP1 mRNA levels were not induced in pNISR8 cells overexpressing IRF1. Similar results were observed after evaluating of other ISG transcripts such as IL7 mRNA.

Intrigued by this result, we decided to evaluate whether these genes could be induced in pNISR8 cells in response to the NF-κB pathway. In fact, many ISGs can be induced by NF-κB, including *IL7, IRF1, GBP1* and IFNα can induce the levels of well-known NF-κB targets such as *CXCL10* or *IL6.* However, this was observed in HeLa but not in pNISR8 cells (**Supplementary Figure 2D**). Similarly, *IRF1, GBP1, CXCL10, IL7, IL13, IL12p35* or *IL12p40* were induced after the expression of NF-κB transcription factor RELA (**Supplementary Figure 3A**) in HeLa but not in pNISR8 cells (**Figure 4C** and **Supplementary Figure 3B**). IFNγ treatment also failed to induce the expression of *GBP1* and *CXCL10* target genes in pNISR8 cells (**Supplementary Figure 3C**).

To determine whether pNISR8 cells also fail to respond properly to antiviral/inflammation-unrelated inducers, we treated HeLa and pNISR8 cells with tunicamycin and induction of the unfolded protein response (UPR) factors *CHOP* and *TRIB3* was evaluated by RT-qPCR. Similar responses were observed in both cells, indicating that pNISR8 cells do not have a general defect in gene induction (**Supplementary Figure 3D**). Interestingly, ISR8 levels are induced by IFN treatment (**Figure 1**) or overexpression of IRF1 or RELA (**Supplementary Figure 4A**) but not by tunicamycin (**Supplementary Figure 4B**). Therefore, there is a correlation between factors that induce ISR8 and those that fail to activate antiviral or inflammatory genes in ISR8-depleted cells.

### pNISR8 Cells Have Functional IFN and NF-κB Signaling Pathways

**Figure 5:**
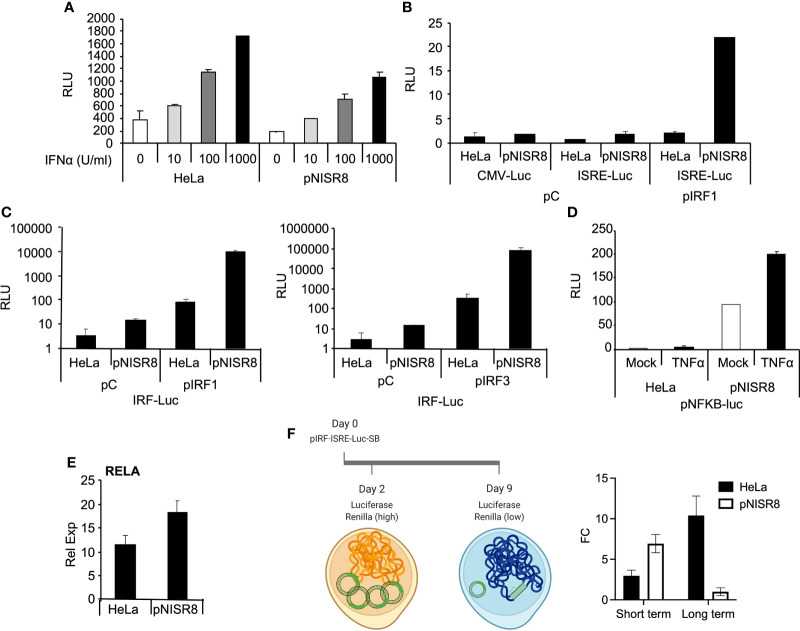
Analysis of the response of episomic or genomic reporter genes in *ISR8*-disrupted cells after IRF1 or RELA overexpression and IFNα treatment. **(A)** Evaluation of relative luciferase units (RLU) in the indicated cells transfected for 48 h with pISRE-Luc and treated with the indicated doses of IFNα. A plasmid expressing renilla´s luciferase was also co-transfected in all cases as a control. **(B)** Similar to A but cells were co-transfected with pCMV-Luc, pISRE-Luc and a control plasmid or pIRF1. **(C)** Similar to C but cells were transfected with pIRF-Luc and pIRF1 or pIRF3. **(D)** Relative luciferase units (RLU) in the indicated cells transfected with pNF-κB-Luc and mock-treated or treated with TNFα for 6 h. **(E)** RELA mRNA was evaluated in HeLa and pNISR8 cells by qRT-PCR and normalized to GAPDH mRNA. **(F)** Schematic of the experiment is shown to the left. The indicated cells were transfected with pSB-IRF·ISRE-Luc and plasmids expressing renilla´s luciferase and the transposase. Forty-eight hours or 9 days after transfection and puromycin selection, luciferase signal was measured and fold change (FC) of RLU in IFNα-treated and untreated cells is shown. Error bars indicate standard deviations. Experiments were performed at least four times and a representative figure is shown.

Given that IFNα treatment or IRF1 overexpression did not induce ISGs in pNISR8 cells, we wondered whether these cells have a functional IFN pathway. STAT1, STAT2 and IRF9 mRNAs were induced in HeLa cells soon after IFNα treatment and in pNISR8 cells at delayed times post-IFNα incubation (**Figure 4D**). These genes must be functional, because IFNα treatment of HeLa or pNISR8 cells transfected with pISRE-Luc (which expresses luciferase after activation of the type I IFN pathway), shows similar luciferase induction in both cells (**Figure 5A**). Together, these results indicate that although IFNα cannot induce the expression of some ISGs in pNISR8 cells, it can induce the expression of exogenous genes and induce delayed expression of certain endogenous ISGs.

We used a similar strategy to determine whether overexpression of IRF1 can induce the expression of luciferase reporters in pNISR8 cells. HeLa and pNISR8 cells were transfected with a control plasmid or pIRF1 and co-transfected with pCMV-Luc as a control; pISRE-Luc, which responds to type I IFN and IRFs; or pIRF-Luc, which responds to IRFs. Luciferase levels from pISRE-Luc and pIRF-Luc plasmids were increased in both cells when pIRF1 was co-transfected (**Figures 5B, C**). Similar results were obtained in cells transfected with a plasmid expressing IRF3 (**Figure 5C**) or by transfection of IRF1 or IRF3 in ex2NISR8 cells (**Supplementary Figure 5**). Similarly, treatment of pNISR8 cells with the NF-κB-inducer TNFα results in increased luciferase expression from a plasmid that expresses luciferase from an NF-κB-inducible promoter (pNF-κB-Luc) (**Figure 5D**). Surprisingly, compared to control HeLa cells, luciferase expression was higher in IRF1 or IRF3-transfected pNISR8 cells (probably related to the high levels of IRF1 in these cells (**Figure 4A**) or in TNFα-treated pNISR8 cells. The latter may result from increased RELA mRNA and protein levels in pNISR8 compared to HeLa cells (**Figure 5E** and see below). In line with these results, the basal expression of *CXCL10* is higher in pNISR8 cells than in HeLa cells (**Supplementary Figure 2D**). In summary, in pNISR8 cells, overexpression of IRF1 or induction of the NF-κB pathway activates the expression of exogenous episomic target genes but fails to increase the expression of endogenous targets.

Surprised by these results, we decided to evaluate what would happen when we insert an IFNα-inducible luciferase gene into the genome. To this aim, we cloned a firefly luciferase reporter with an IFN/IRF1 responsive promoter into a sleeping beauty transposon backbone that allows selection with puromycin. Then, we transfected this plasmid, a vector expressing the sleeping beauty transposon and a control plasmid with the Renilla luciferase gene under a ubiquitous promoter. As expected, two days after transfection of HeLa and pNISR8 cells, we observed a high increase in firefly luciferase levels after 6h of IFNα treatment (**Figure 5F**). This time represents the effect observed over the episomic vectors, as Renilla luciferase levels in these cells were high. Instead, nine days after transfection, Renilla luciferase levels were close to background, indicating that most of the episomic vector had been lost. At this time, when most firefly luciferase signal should come from the genome-integrated gene, we observed a high induction after IFNα treatment in HeLa cells but not in pNISR8 cells. We then concluded that IFN response is active in pNISR8 cells and induces the expression from episomic vectors, but fails to activate endogenous or exogenous target genes integrated into the genome.

### Analysis of Gene Silencing Blockade in pNISR8 Cells

**Figure 6:**
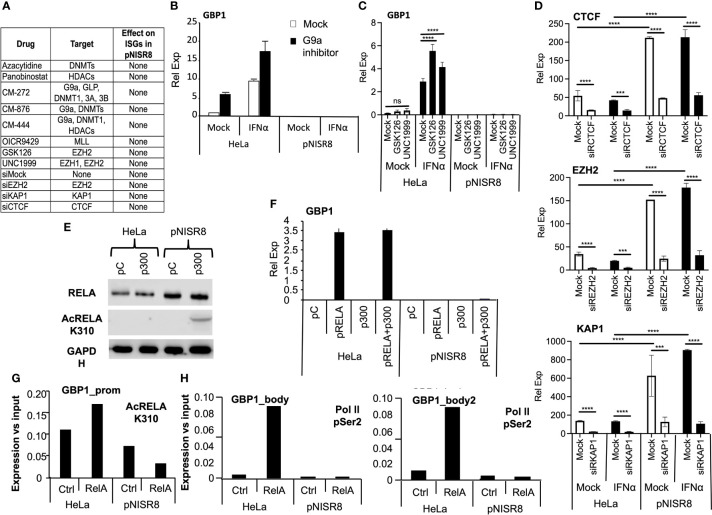
Effect of silencing inhibitors or enhancer overexpression in *ISR8*-disrupted cells. **(A)** Table with all the inhibitors tested, their targets and their effect over ISG levels in IFNα treated pNISR8 cells. **(B, C)** HeLa and pNISR8 cells were mock-treated, incubated with IFNα, and treated with G9a inhibitors **(B)** or the indicated EZH2 inhibitors. GBP1 mRNA levels were evaluated by qRT-PCR. **(D)** HeLa and pNISR8 cells were transfected with control siRNAs (Mock) or siRNAs targeting CTCF, EZH2 or KAP1. Six hours prior harvest, cells were treated or not with IFNα. Forty-eight hours after transfection CTCF, EZH2 or KAP1 mRNA levels were evaluated by qRT-PCR. **(E)** HeLa cells were transfected with a control plasmid (pC) or a plasmid expressing p300 and 48h later, total and acetylated RELA was evaluated by Western-blot. GAPDH was also visualized as a loading control. **(F)** HeLa and pNISR8 cells were transfected with a control plasmid (pC), a plasmid expressing p300 and/or pRELA. Forty-eight hours later GBP1 mRNA levels were quantified by qRT-PCR. GAPDH mRNA was also evaluated and used as a reference. **(G, H)** HeLa and pNISR8 cells were transfected with a control plasmid (Ctrl) or a plasmid expressing RELA. Forty-eight hours later ChIP experiments were performed with anti- K310 acetylation RELA **(G)** or anti Ser2 phosphorylation Pol II antibodies **(H)**. Immunoprecipitated DNA was PCR amplified with primers from the GBP1 promoter **(G)** or the gene body **(H)**. Error bars indicate standard deviations. Experiments were performed at least twice and a representative figure is shown. Statistical analysis is shown for relevant images.

We hypothesized that pNISR8 cells do not respond properly to STATs, IRF1 or RELA because ISG/NF-κB promoters have repressive chromatin marks (H3K27me3 and H3K9me3) or poor levels of active chromatin marks (H3K4me3 and H3K27ac). Several works show that deacetylase or methyltransferase repressors favor type I IFN and NF-κB responses, and that these pathways can be silenced by KAP1 or CTCF binding at promoters of target genes (45–49). Therefore, we treated HeLa and pNISR8 cells with the IC50 of small molecules that inhibit histone deacetylases (HDAC; Panovinostat), DNA methyltransferases (DNMT; Azacytidine), polycomb repressive complex (PRC) essential subunit (EZH1/2; GSK126 or UNC1999), G9a histone methyltransferase (HMT G9a; CM272) alone or in combination (G9a +DNMT, G9a +HDAC inhibitors), or with siRNAs against repressive molecules EZH2, KAP1 or CTCF (**Figure 6A**). We confirmed the functionality of silencing suppressors by measuring the expression of endogenous retroviruses whose transcription is normally repressed in heterochromatic regions (**Supplementary Figure 6**) (50, 51). In fact, re-induction of endogenous retroviruses by silencing suppressors led to activation of IFN response and increased levels of ISGs in HeLa cells (**Figure 6B**) (52). However, none of the inhibitors tested affected the expression of *GBP1* in IFNα-treated or untreated pNISR8 cells (**Figure 6A**–**C**). Similar results were observed after efficient inhibition of CTCF, KAP1 or EZH2 mRNAs using siRNAs (**Figures 6A, D**). Instead, we observed that their levels are higher in pNISR8 than in HeLa cells (**Figure 6D**).

Given the negative results obtained by blocking silencing, we tried to favor H3K27 acetylation by overexpression of p300 histone acetylase. Interestingly, p300 also acetylates RELA to increase its activity. Transfection of a plasmid expressing p300 increased the levels of acetylated RELA in pNISR8 cells (**Figure 6E**). However, overexpression of p300, RELA or both in pNISR8 cells failed to induce the expression of RELA targets *CXCL10* or *GBP1* (**Figure 6F** and **Supplementary Figure 7A**). In agreement with this, chromatin immunoprecipitation (ChIP) using RELA K310 acetylation antibodies shows lower levels of this protein in *GBP1* and *CXCL10* promoters of pNISR8 than in HeLa cells (**Figure 6G** and **Supplementary Figure 7B**). ChIP using Ser2 phosphorylated polymerase II antibodies also shows decreased levels of this factor in the *GBP1* gene body of pNISR8 cells transfected with pRELA than in HeLa cells (**Figure 6H**).

### Enhancer Function of *ISR8* Locus

**Figure 7:**
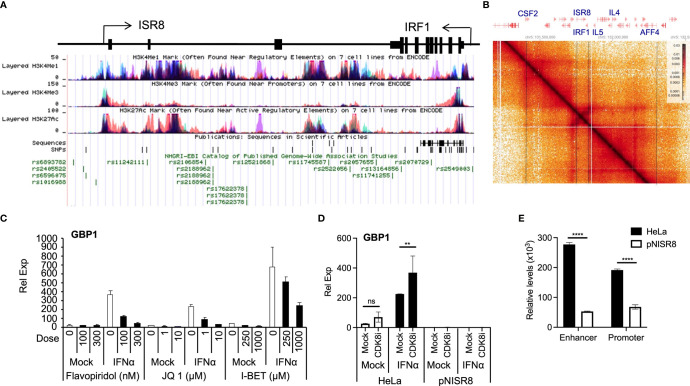
Analysis of enhancer function in IFNα-treated cells. **(A)** Schematic of *ISR8* locus from UCSC showing *ISR8* and *IRF1* genomic region, transcription, enrichment of H3K27ac, H3K4me1 and H3K4me3 marks and SNPs (https://genome.ucsc.edu/). **(B)** HiC image showing the interactions observed around the *ISR8* and *IRF1* genomic region obtained by HiGlass. Representative genes are highlighted at the top and intermittent lines are used to mark their position in the image. **(C, D)**. The indicated cells were mock-treated or treated with JQ1, flavopiridol, I-BET1 **(C)** or the CDK8 inhibitor CCT251545 **(D)**. Then, 0 or 1000U/ml of IFNα were added for 6h and GBP1 mRNA levels were measured by qRT-PCR. GAPDH mRNA was also evaluated and used as a reference. Experiments were performed at least twice and a representative figure is shown. **(E)** FAIRE was performed with HeLa and pNISR8 cells and chromatin accessibility was evaluated at the ISR8 enhancer and promoter regions with high H3K4me1 levels. The experiment was performed twice and the average of the results is shown. Error bars indicate standard deviations. Statistical significance is shown where relevant.

Transcription requires proper enhancer function. ENCODE ChIP data show that the *ISR8* region is covered by H3K27ac and H3K4me1 and has low levels of H3K4me3, indicating that the *ISR8* sequence has enhancer marks (**Figure 7A**). Fifty-six SNPs have been described in the region, many of which associate with asthma and respiratory diseases (13), eczema or hay fever (3), chronic inflammatory diseases (3) such as Crohn’s disease (5), inflammatory bowel disease (2), ulcerative colitis (2), juvenile idiopathic arthritis, and others such as pediatric autoimmune diseases or multiple sclerosis (**Figure 7A** and **Supplementary Table 2**). Public HiC data show that *ISR8* and *IRF1* are in the same TAD and that this could be enlarged to include neighbor TADs that contain cytokine-expressing genes such as the colony stimulator factor 2 (CSF2), IL4 or IL5 (**Figure 7B**).

Thus, the phenotypes observed in pNISR8 cells could be related to a defect in the enhancer function of the *ISR8* locus. Indeed, we have confirmed that relevant enhancer factors such as BRD4, CDK8 or CDK9 (53) are required for ISG induction after IFNα-treatment of HeLa cells. GBP1 and CXCL10 mRNA levels are not induced in cells treated with flavopiridol (CDK9 inhibitor), JQ1 and I-BET BRD4 bromodomain inhibitors or CCT251545 (CDK8 inhibitor) (54) (**Figures 7C**, **D**, **Supplementary Figures 8A, B**). Tunicamycin induction of TRIB mRNA also failed in HeLa or pNISR8 cells treated with CDK9 or bromodomain inhibitors (**Supplementary Figure 8C**). In addition, when we measure chromatin accessibility in the ISR8 region using FAIRE (55) we find a strong decrease in *ISR8* promoter and enhancer regions in pNISR8 compared to HeLa cells (**Figure 7E**).

### Several Zinc Finger Proteins Are Highly Upregulated in ISR8-Disrupted Cells

Enhancers may control the expression of genomic regions located in the vicinity, both in the same chromosome or the same chromosome territory. Thus, ISR8-enhancer disruption may affect IRF1 expression as both are located in the same TAD (**Figure 7B**). To search for additional ISR8-regulated factors, we compared the transcriptomes of HeLa and pNISR8 cells treated or not with IFNα. The analysis of the results indicated that HeLa and pNISR8 transcriptomes are located far in the PCA space (**Supplementary Figure 9**). Interestingly, while IFNα treatment impacts HeLa transcriptome, it does not alter pNISR8 cells. Analysis of coding genes showed 1672 genes upregulated (FC>5) and 472 genes downregulated in pNISR8 versus IFNα-treated HeLa cells (**Supplementary Table 3**). Downregulated genes are highly associated with type I IFN signaling and response and extracellular matrix organization, while upregulated genes are enriched for those regulated by EZH2 (p-value = 2,5E-16) and SUZ12, another PRC2 component (p-value = 4,5E-6), according to ENCODE ChIP-Seq data, and for biological processes and molecular functions related to the regulation of transcription at the DNA and RNA levels (**Supplementary Figures 10A, B**). Prominent among these factors are 110 Zinc finger proteins (ZNF) (**Supplementary Figure 10C**). We were also surprised by the levels of XIST lncRNA, which is barely detected in HeLa cells and expressed to similar levels as mitochondrial genes in pNISR8 cells. The highly significant upregulation of XIST and several ZNFs was confirmed by RT-qPCR in pNISR8 and in ex2NISR8 cells (**Supplementary Figures 10D, E**).

## Discussion

We have previously identified ISR8/IRF1-AS1 as a lncRNA induced at early times post-IFNα treatment (27). Here we show that IFNα increases the expression of ISR8 mRNA in cell lines and primary cells (**Figure 1**). Interestingly, ISR8 is also induced by IRF1 and RELA, suggesting that the *ISR8* locus may play a role in antiviral and inflammatory processes (**Supplementary Figure 4A**). To determine ISR8 function, we disrupted the *ISR8* locus in HeLa and Hap 1 cells using the CRISPR-Cas system (**Figure 2** and **Supplementary Figure 2**). We failed to isolate cells with a complete *ISR8* deletion, suggesting that this region might be essential for cell viability. Instead, we obtained clones with a neomycin or a puromycin resistance gene followed by polyadenylation sequences inserted after the promoter (pNISR8) or in exon 2 (ex2NISR8) of the *ISR8* locus (**Figure 2A**). In pNISR8 cells, transcription from the *ISR8* promoter should transcribe the resistance sequences and stop at the poly-A sequences. In ex2NISR8 cells, transcription from the *ISR8* promoter should result in a chimeric ISR8 mRNA with the first exon and the initial nucleotides of the second exon fused to the neomycin resistance sequences. In fact, we failed to detect ISR8 mRNAs by RT-qPCR in IFNα-treated pNISR8 and ex2NISR8 cells using oligonucleotides corresponding to the initial nucleotides of exon 2 and exon 3 (**Figure 2B**). The neomycin insertion downstream or the expression of the chimeric transcript may be relevant for the milder phenotype observed in ex2NISR8 cells compared to pNISR8 cells (**Figure 2F**).

Several independent clones of pNISR8 and ex2NISR8 show a defective IFNα response, indicating that this is due to *ISR8* locus disruption rather than random insertions or deletions generated by Cas9 in other regions of the genome (**Figures 2C, D** and **Supplementary Figures 2A–C**). Interestingly, *ISR8*-disrupted cells show a defective response to IFNα, IFNγ, and NF-κB, pathways that are strongly related to antiviral and inflammatory responses, but not to inducers of UPR (**Figure 2** and **Supplementary Figures 3**, **10**). Thus, a general mechanism of induction is not affected in these cells. Furthermore, overexpression of transcription factors such as IRF1 and RELA fail to induce the expression of ISGs and NF-κB targets in pNISR8 cells (**Figure 4** and **Supplementary Figure 3**). In fact, RELA and NF-κB signaling are increased in these cells compared to HeLa controls (**Figures 5D–E**, **6E**) and this contributes to the increased levels of IRF1 observed in pIRF1-transfected pNISR8 cells. Other cellular factors involved in gene silencing and chromatin dynamics such as EZH2, KAP1 and CTCF are also increased in the cells edited at *ISR8* locus compared to HeLa cells (**Figure 6D**). KAP1 is a transcriptional repressor recruited to the DNA by binding to KRAB domains of Zn finger protein transcription factors. In addition, KAP1 association with STAT1 inhibits IRF1 mRNA and the expression of its target genes, most likely *via* HDACs (47, 48). CTCF binds DNA to mediate long-range interactions between DNA regions and allows the formation of TADs, important in enhancer-promoter interactions. However, CTCF can also act as a negative regulator by binding to IFNα and IFNβ gene promoters and blocking their transcription (49, 56). EZH2 is also a well-known negative regulator of immune and antiviral genes (57, 58). However, downregulation of EZH2, KAP1 or CTCF in pNISR8 cells does not allow a normal expression of IFNα and NF-κB targets after their induction (**Figure 6D** and data not shown). These results suggest that increased RELA, EZH2, KAP1 and CTCF expression in pISR8 cells may be a consequence rather than the cause of the altered regulation observed in *ISR8*-disrupted cells. Interestingly, despite the high levels, EZH2 seems non-functional in pNISR8 cells, as genes upregulated in these cells versus HeLa controls are highly enriched in EZH2-target genes.

While EZH2 compacts chromatin by writing H3K27me3, G9a is another histone methyltransferase (HMT) that represses gene expression with H3K9me3 marks. G9a has also been shown to repress ISG expression (52). In addition to these histone marks, DNA methylation is also associated with gene repression (59, 60). Therefore, repressive epigenetic marks are deposited by the coordinated action of certain HMTs, DNMTs and HDACs. However, inhibition or downregulation of HDACs, DNMTs or HMTs in pNISR8 cells failed to recover proper IFN induction in these cells (**Figures 6A–C**). Similar results were obtained after upregulation of the p300 acetyl-transferase activator (**Figures 6E, F**).

Rather than enhanced silencing, we believe that an enhancer defect causes the deficient response to IFNα and NF-kB activation in pNISR8 cells. Enhancers are essential to induce poised promoters and fire polymerase II elongation (61). This allows a fast response to critical stimuli such as type I IFN or TNFα. We show that: (i) enhancer function is essential for RELA and IFNα stimulation of target genes (62) (**Figures 7C, D** and **Supplementary Figure 8**); (ii) target gene promoters in pNISR8 cells show low levels of acetylated RELA (**Figure 6G**); (iii) target genes in pNISR8 cells show decreased levels of pol II Ser2 phosphorylation, a marker for elongation (**Figure 6H**); (iv) induction defects in pNISR8 cells are observed in genomic but not episomic genes (**Figure 5**); (v) *ISR8* gene has enhancer marks according to ChIP experiments performed by ENCODE (**Figure 7A**) and (vi) this region turns inaccessible in pNISR8 cells according to FAIRE (**Figure 7E**).

Considering all these results, we hypothesize that genome editing in pNISR8 cells could have led to chromatin compaction of the region and defects in enhancer function at the *ISR8* locus (**Figure 7**), in addition to affecting ISR8 expression (**Figure 2B**). Note that enhancers can act as operative transcriptional units that transcribe for eRNAs. This transcription has been suggested as a byproduct of polymerase II binding to the enhancer region; however, some eRNAs mediate in the enhancer function. In some cases, only the act of enhancer transcription is required for enhancer function, while in others, eRNA transcripts are essential for enhancer function. Indeed, some eRNAs favor DNA looping by binding to Mediator complex or cohesion, as is the case of Kallikrein-related peptidase 3 (KLK3) eRNA and ncRNA-a7, or NRIP1 eRNA respectively (20, 21, 63). However, *ISR8* does not fulfill the general characteristics of most eRNAs, as ISR8 is an abundant transcript spliced and polyadenylated (19, 63). Inhibition of *ISR8* with gapmers that target intronic regions should degrade ISR8 RNA precursors co-transcriptionally. HeLa cells treated with these gapmers do not show any defect in ISG expression after IFNα treatment, suggesting that *ISR8* transcription is not required or that transcription of just few nucleotides could be enough for enhancer functionality. In the same line, cells disrupted in the *ISR8* locus to introduce a cassette have to allow transcription initiation to express the resistance gene. However, pNISR8 and ex2NISR8 cells do not induce ISG expression, suggesting that the mere act of transcription in the *ISR8* region is insufficient for *ISR8* enhancer function. In turn, our current hypothesis is that activation of the *ISR8* enhancer after a trigger with IFNα or NF-κB induction leads to binding of *ISR8* enhancer to specific promoters and transcription initiation complexes, which allow *ISR8* transcription. Then, *ISR8* transcription marks enhancer functionality and may serve as a readout for the activity of the enhancer located at the *ISR8* locus. Therefore, *ISR8*-disrupted cells respond properly to the induction of the UPR pathway, which does not induce *ISR8* expression, while they fail to respond to IFNα and NF-κB pathways, which increase ISR8 levels in HeLa cells. Further experiments will be performed to address the enhancer function of *ISR8*. The region with enhancer marks is close to 60 megabases, being a good candidate for Cap-STARR-Seq (Capture self-transcribing active regulatory regions sequencing) (64). This would provide a direct functional and quantitative readout of the enhancer activity of ISR8 loci.

Enhancers control the expression of genes located nearby. Interestingly, out of the genes studied in this work, *IRF1, IL12p40* and *IL13* are located close to *ISR8*, while the other ISGs are in distant regions of the genome. Transcriptome analysis of *ISR8* region in pNISR8 cells, compared to HeLa controls, shows none or low levels of ISR8 and IRF1, as expected, decreased SLC22A4, and increased SOWAHA and PDLIM4 (**Supplementary Table 3**). None of the latter has been related to type I IFN or TNFα activity. 4C experiments seem insufficient to determine all the regular partners of the *ISR8* enhancer in IFNα-treated cells. A scrupulous analysis of public 4C data shows that the *ISR8* region is in close spatial proximity to sequences within the same chromosome belonging to the same TAD (**Figure 7B**). Conversely, interactions between the *ISR8* region and other ISGs are not detected. In fact, close proximity is not required for promoter activation by enhancers as they can form phase-separated condensates of activators (65). Techniques that provide 3D proximity data or SPRITE-like technology may be required to have a more comprehensive picture of *ISR8* enhancer partners, as SPRITE crosslinks DNA and RNA to identify components of specific chromosome territories (66, 67). Using this technology, it has been shown how X-inactive specific transcript (XIST) lncRNA transcription allows seeding of regulatory factors that amplify the initial signal and permit the regulation of wider genomic regions (65). A similar mechanism could also function for ISR8 regulation. In fact, ISR8 depletion leads to an extraordinary upregulation of XIST (**Supplementary Figure 10E**) that could lead to a broader silencing, including that of antiviral and inflammatory genes. Note that the X chromosome silenced by XIST contains many immunity-related genes and that they are silenced by XIST after development (68, 69). Therefore, XIST deregulation leads to autoimmune diseases in females, representing 80% of the people affected by these diseases (70).

Alternatively, the *ISR8* enhancer may control the expression of one or several intermediate factors that function as master regulators of ZNFs or XIST expression. Thus, the levels of many ZNFs are highly upregulated in ISR8 disrupted cells (**Supplementary Figures 10C, D**). ZFNs belong to one of the largest families of proteins in mammalian cells, partly due in to their high sensitivity to evolutionary pressure. Notably, KAP1 bound ZFN proteins co-evolve with transposable elements and lead to their silencing to guarantee genome stability (71, 72). In addition to transcription factors and depending on the array of Zn finger domains, ZFN can also bind and regulate RNA, proteins or lipids. Interestingly, they have been described to regulate the immune response at transcriptional and post-trancriptional levels [reviewed in (73)]. Therefore, KAP1 inhibition is not sufficient to kill all the regulatory potential of ZFNs, as we have shown (**Figure 6D**). Open questions are whether ZFNs silence IFNα and TNFα genes in ISR8 disrupted cells and the mechanisms that link ISR8 with ZFN overexpression.

Independently of the ISR8 mechanism of action, our results clearly show how genome editing with CRISPR-Cas may lead to local genomic alterations with widespread effects. In addition, SNPs associated with several inflammatory and autoimmune diseases have been described in the *ISR8* region. These diseases include Crohn’s disease, inflammatory bowel disease, ulcerative colitis and asthma, where the response to IFN and inflammatory cytokines is excessive and leads to damage of the affected tissues by these cytokines (71–76) (**Supplementary Table 2**). In conjunction with our results, this suggests that the *ISR8* region is relevant to control the expression of ISGs and pro-inflammatory molecules and that the *ISR8* locus could be a target for therapies against inflammatory diseases.

## Data Availability Statement

The data presented in the study are deposited in the NCBI GEO repository, accession number GSE205276; https://www.ncbi.nlm.nih.gov/geo/query/acc.cgi?acc=GSE205276.

## Ethics Statement

The studies involving human participants were reviewed and approved by Comité etico de la Investigación de la Universidad de Navarra. The patients/participants provided their written informed consent to participate in this study.

## Author Contributions

MB, LP-M and JU: conceptualization, formal analysis, investigation, methodology, visualization, writing. NR and CP: investigation. PF: conceptualization, formal analysis, funding acquisition, methodology, project administration, resources, supervision, visualization, writing.

## Funding

This work was supported by grants SAF2015-70971-R and RTI2018-101759-BI00 finance by MCIN/AEI /10.13039/501100011033 and by FEDER Una manera de hacer Europa to P.F. Scientific Foundation of the Spanish Association Against Cancer (AECC IDEAS20169FORT to PF) and by the Instituto de Salud Carlos III, which finances the Centro de Investigación Biomédica en Red de Enfermedades Hepáticas y Digestivas (CIBEREhd). MB was a recipient of a FPI fellowship, JU of a University of Navarra´s Asociación de Amigos fellowship and LP-M is a recipient of a PFIS fellowship (FI20/00074) by the National Institute of Health Carlos III and FSE “Investing in Your Future”.

## Conflict of Interest

The authors declare that the research was conducted in the absence of any commercial or financial relationships that could be construed as a potential conflict of interest.

## Publisher’s Note

All claims expressed in this article are solely those of the authors and do not necessarily represent those of their affiliated organizations, or those of the publisher, the editors and the reviewers. Any product that may be evaluated in this article, or claim that may be made by its manufacturer, is not guaranteed or endorsed by the publisher.

## Supplementary Material

Supplementary Figure 1 Genomic analysis of the clones obtained by CRISPR-Cas9 technique. Schematic of the position of the primers used for PCR analysis of pNISR8 **(A)** and ex2NISR8 **(B)** and the result of the PCR amplification after electrophoresis in agarose gels. The distance between each set of primers and the sizes of the amplified products are indicated. Arrows show the position of the indicated size according to molecular weight markers.

Supplementary Figure 2 Analysis of the induction of ISGs and inflammatory genes in pNISR8 clones. **(A)**. IL7 mRNA levels in HeLa and three independent clones of pNISR8 treated with 0 or 1000 U/ml IFNα for 6 h. **(B)**. GBP1 mRNA levels of Hap1 and two independent clones of Hap-derived pNISR8 and pPISR8 cells treated with 0 or 1000 U/ml IFNα for 6 h. **(C)**. GBP1 mRNA levels of Hap1 and two independent clones of Hap-derived pNISR8 cells transfected with a control plasmid (Mock) or pISR8 and treated with 0 or 1000 U/ml IFNα for 6 h. **(D)**. CXCL10 and IL6 mRNA levels in HeLa or pNISR8 cells treated with 0 or 1000 U/ml of IFNα for the indicated time. GAPDH mRNA was also evaluated and used as a reference. Error bars indicate standard deviations. Experiments were performed at least three times and a representative figure is shown.

Supplementary Figure 3 Analysis of NF-κB and IFNγ pathways and unfolded protein response (UPR) in pNISR8 cells. **(A, B)**. Levels of the indicated mRNAs in HeLa and pNISR8 cells transfected with a control plasmid (pC) or pRELA for 48h and treated with 0 or 1000 U/ml of IFNα for 6h. **(C)**. Levels of GBP1 and CXCL10 mRNAs in the indicated cells treated or not with IFNγ for 6 h. **(D)**. Levels of CHOP and TRIB3 mRNAs in the indicated cells treated or not with tunicamycin for the indicated times. GAPDH mRNA was also evaluated and used as a reference. Error bars indicate standard deviations. Experiments were performed at least twice and a representative figure is shown.

Supplementary Figure 4 Analysis of ISR8 induction by RELA and/or IRF1 overexpression or tunicamycin treatment. **(A, B)**. ISR8 mRNA levels were evaluated in HeLa cells transfected with a control plasmid (pC), pRELA and/or pIRF1 **(A)** or treated with tunicamycin for the indicated times **(B)**. GAPDH mRNA was also evaluated and used as a reference. Error bars indicate standard deviations. Experiments were performed at least twice and a representative figure is shown.

Supplementary Figure 5 Analysis of IRF-Luc expression after IRF1 or IRF3 overexpression in ex2NISR8 cells. HeLa and ex2NISR8 cells were co-transfected with pIRF-Luc and with a control plasmid (pC), pIRF1 or pIRF3. A plasmid expressing renilla´s luciferase was also co-transfected in all cases as a control. Forty-eight hours later luciferase signal was measured and relative luciferase units (RLU) were plotted. Error bars indicate standard deviations. Experiments were performed twice. Statistical analysis is shown.

Supplementary Figure 6 Analysis of the functionality of G9a inhibitors. HeLa cells were mock-treated or treated with G9a inhibitors and ERV MTL2B4 RNA expression was evaluated by qRT-PCR. GAPDH mRNA was also evaluated and used as a reference. Error bars indicate standard deviations.

Supplementary Figure 7 Analysis of RELA and p300 overexpression over CXCL10 gene. **(A, B)**. HeLa and pNISR8 cells were transfected with a control plasmid (pC), a plasmid expressing p300 **(A)** and/or pRELA. Forty-eight hours later GBP1 mRNA levels were quantified by qRT-PCR (A) or ChIP experiments were performed with anti- K310 acetylation RELA antibodies **(B)**. Immunoprecipitated DNA was PCR amplified with primers from the CXCL10 promoter. Error bars indicate standard deviations. Experiments were performed at least twice and a representative figure is shown.

Supplementary Figure 8 Analysis of enhancer dependence of CXCL10 activation and UPR response. **(A)**. CXCL10 mRNA levels in HeLa cells mock-treated or treated with the indicated doses of flavopiridol JQ1, I-BET1 and 0 or 1000U/ml of IFNα for 6h. **(B)**. Similar to A but HeLa and pNISR8 cells were evaluated after treatment with the CDK8 inhibitor CCT251545. **(C)**. TRIB3 mRNA levels of the indicated control cells or cells treated with JQ1 and I-BET1 inhibitors with or without tunicamycin. GAPDH mRNA was also evaluated and used as a reference. Error bars indicate standard deviations. Experiments were performed at least twice and a representative figure is shown.

Supplementary Figure 9 PCA of HeLa and pNISR8 cells treated or not with IFNα.

Supplementary Figure 10 Transcriptome analysis of HeLa and pNISR8 cells. **(A)**. Expression levels in the indicated cells of ISGs upregulated over seven-fold in HeLa cells after IFNα treatment. **(B)**. Gene ontology enrichment of biological processes and molecular functions of genes downregulated or upregulated (FC>5) in pNISR8 cells versus IFNα-treated HeLa cells. **(C)**. mRNA levels of Zinc finger proteins upregulated (FC>5) in pNISR8 cells versus HeLa cells in the indicated cells. Only 1/3 of the names are indicated to the left for clarity. **(D, E)**. RNA was isolated from HeLa, pNISR8 and ex2NISR8 cells and the levels of the indicated ZNF transcripts **(D)** or XIST **(E)** were evaluated by qRT-PCR. GAPDH mRNA was also evaluated and used as a reference. Experiments were performed three times and a representative image is shown. Statistical analysis is indicated.

Supplementary Table 1 List of sequences for primers, guides and inhibitors used in this study.

Supplementary Table 2 SNPs related to autoimmune diseases found in *ISR8* loci.

Supplementary Table 3 List of coding genes deregulated in pNISR8 compared to HeLa cells.
