# Supplementary figures and images for: ISR8/IRF1-AS1 Is Relevant for IFNα and NF-κB Responses
Source: Front Immunol. 2022 Jul 4;13:829335. doi: 10.3389/fimmu.2022.829335 (PMC9289242; doi:10.3389/fimmu.2022.829335)

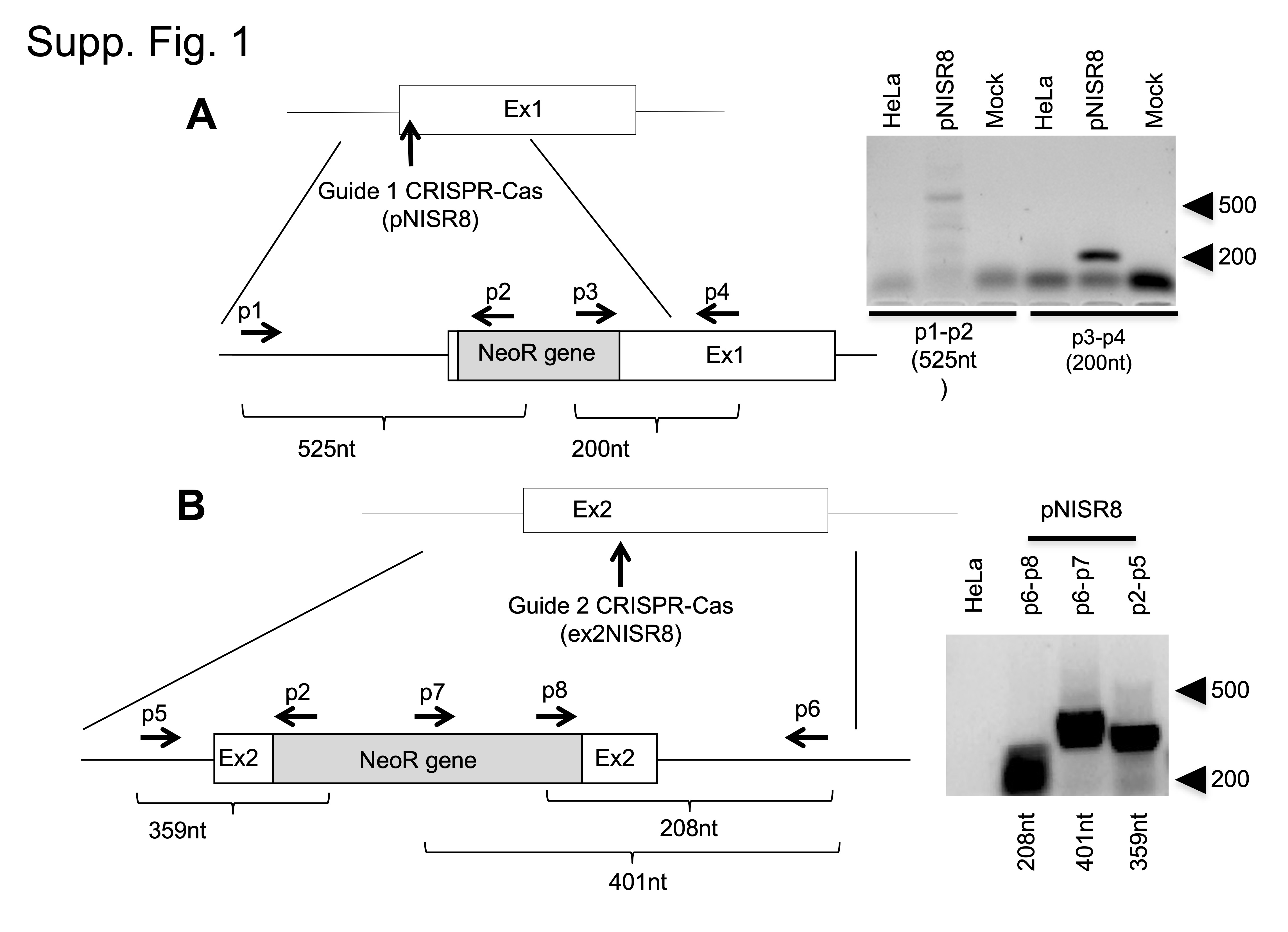

Supplement: Supplementary Figure 1 — Genomic analysis of the clones obtained by CRISPR-Cas9 technique. Schematic of the position of the primers used for PCR analysis of pNISR8 (A) and ex2NISR8 (B) and the result of the PCR amplification after electrophoresis in agarose gels. The distance between each set of primers and the sizes of the amplified products are indicated. Arrows show the position of the indicated size according to molecular weight markers. [file Image_1.jpg]

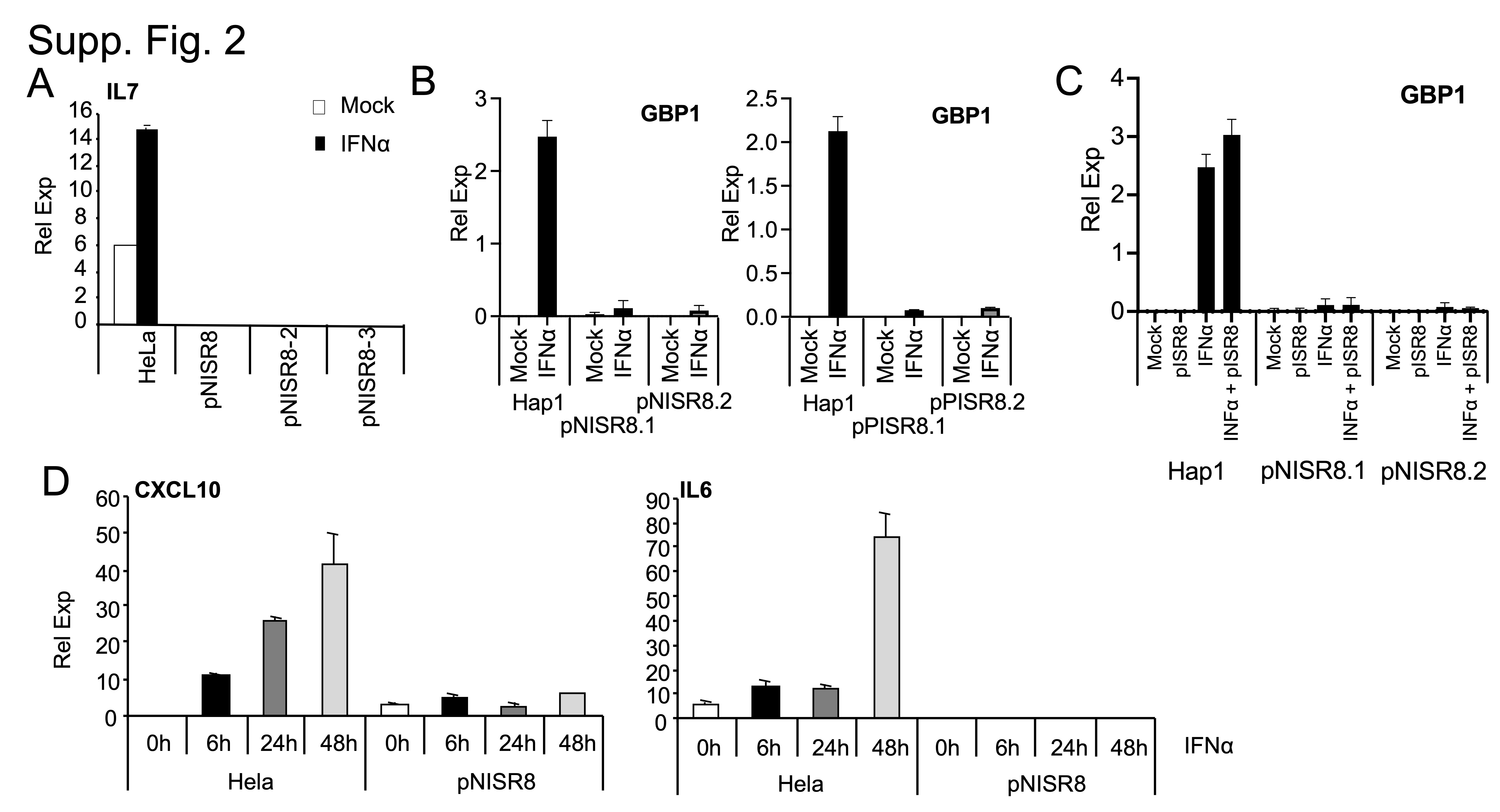

Supplement: Supplementary Figure 2 — Analysis of the induction of ISGs and inflammatory genes in pNISR8 clones. (A). IL7 mRNA levels in HeLa and three independent clones of pNISR8 treated with 0 or 1000 U/ml IFNα for 6 h. (B). GBP1 mRNA levels of Hap1 and two independent clones of Hap-derived pNISR8 and pPISR8 cells treated with 0 or 1000 U/ml IFNα for 6 h. (C). GBP1 mRNA levels of Hap1 and two independent clones of Hap-derived pNISR8 cells transfected with a control plasmid (Mock) or pISR8 and treated with 0 or 1000 U/ml IFNα for 6 h. (D). CXCL10 and IL6 mRNA levels in HeLa or pNISR8 cells treated with 0 or 1000 U/ml of IFNα for the indicated time. GAPDH mRNA was also evaluated and used as a reference. Error bars indicate standard deviations. Experiments were performed at least three times and a representative figure is shown. [file Image_2.jpg]

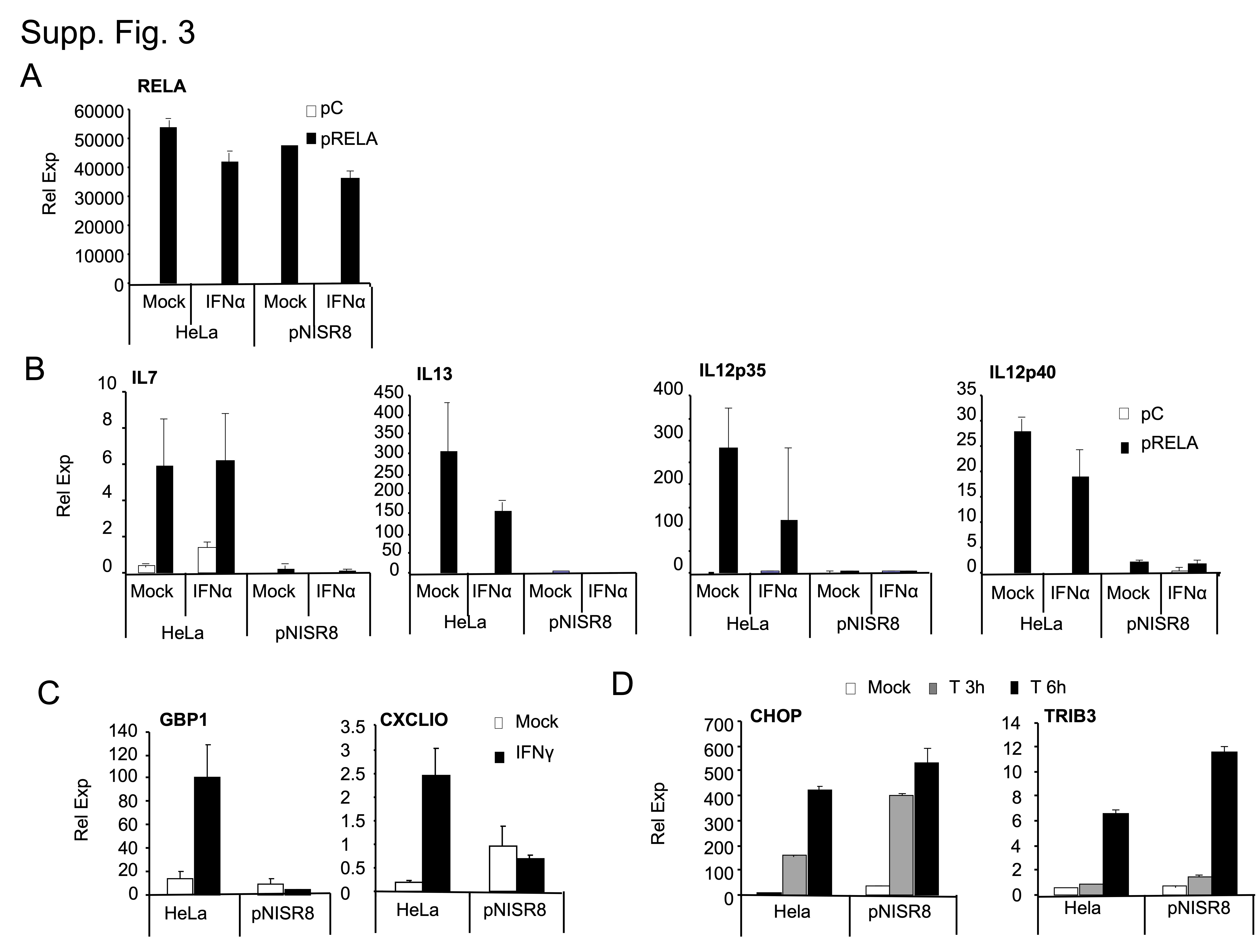

Supplement: Supplementary Figure 3 — Analysis of NF-κB and IFNγ pathways and unfolded protein response (UPR) in pNISR8 cells. (A, B). Levels of the indicated mRNAs in HeLa and pNISR8 cells transfected with a control plasmid (pC) or pRELA for 48h and treated with 0 or 1000 U/ml of IFNα for 6h. (C). Levels of GBP1 and CXCL10 mRNAs in the indicated cells treated or not with IFNγ for 6 h. (D). Levels of CHOP and TRIB3 mRNAs in the indicated cells treated or not with tunicamycin for the indicated times. GAPDH mRNA was also evaluated and used as a reference. Error bars indicate standard deviations. Experiments were performed at least twice and a representative figure is shown. [file Image_3.jpg]

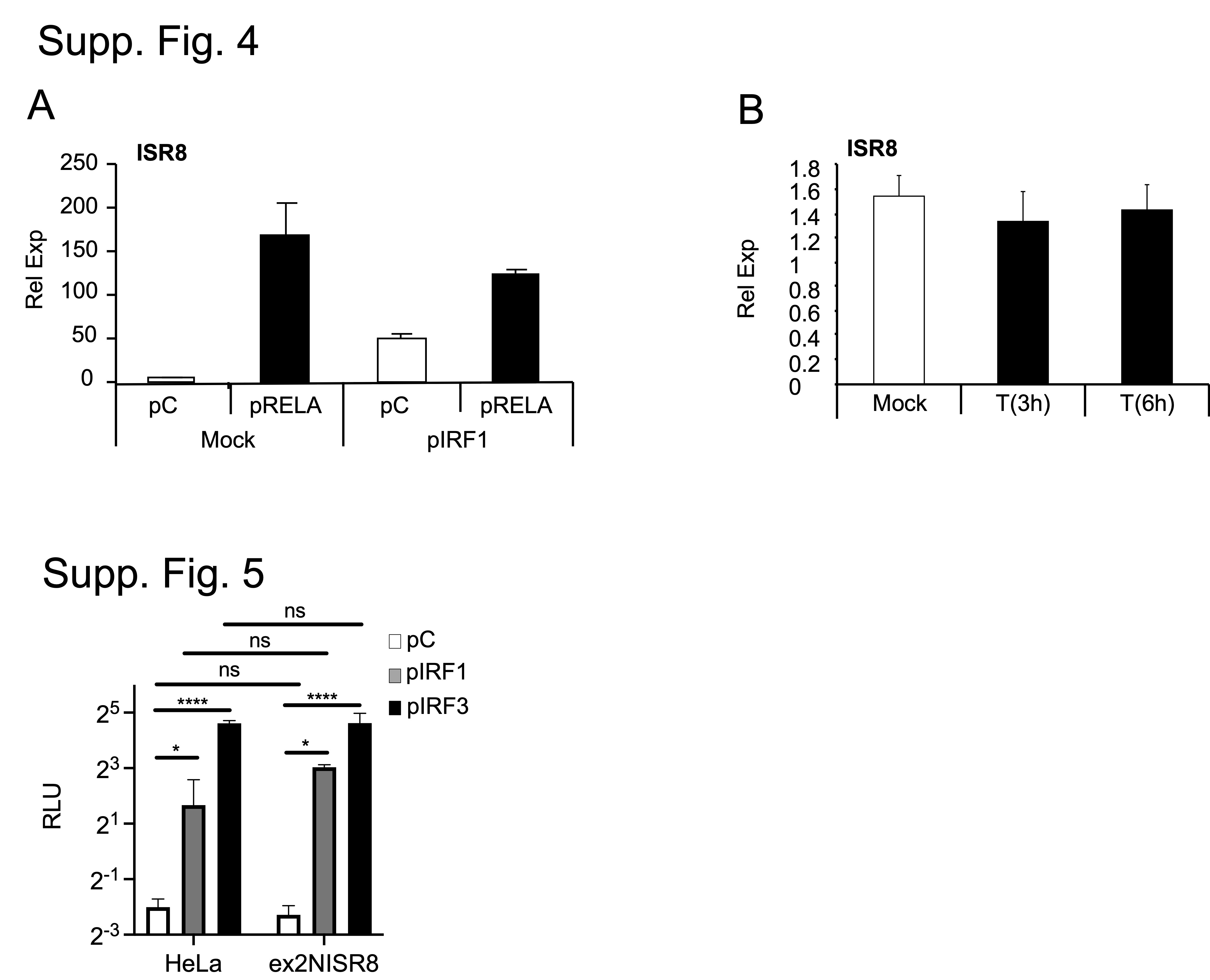

Supplement: Supplementary Figure 4 — Analysis of ISR8 induction by RELA and/or IRF1 overexpression or tunicamycin treatment. (A, B). ISR8 mRNA levels were evaluated in HeLa cells transfected with a control plasmid (pC), pRELA and/or pIRF1 (A) or treated with tunicamycin for the indicated times (B). GAPDH mRNA was also evaluated and used as a reference. Error bars indicate standard deviations. Experiments were performed at least twice and a representative figure is shown. [file Image_4.jpg]

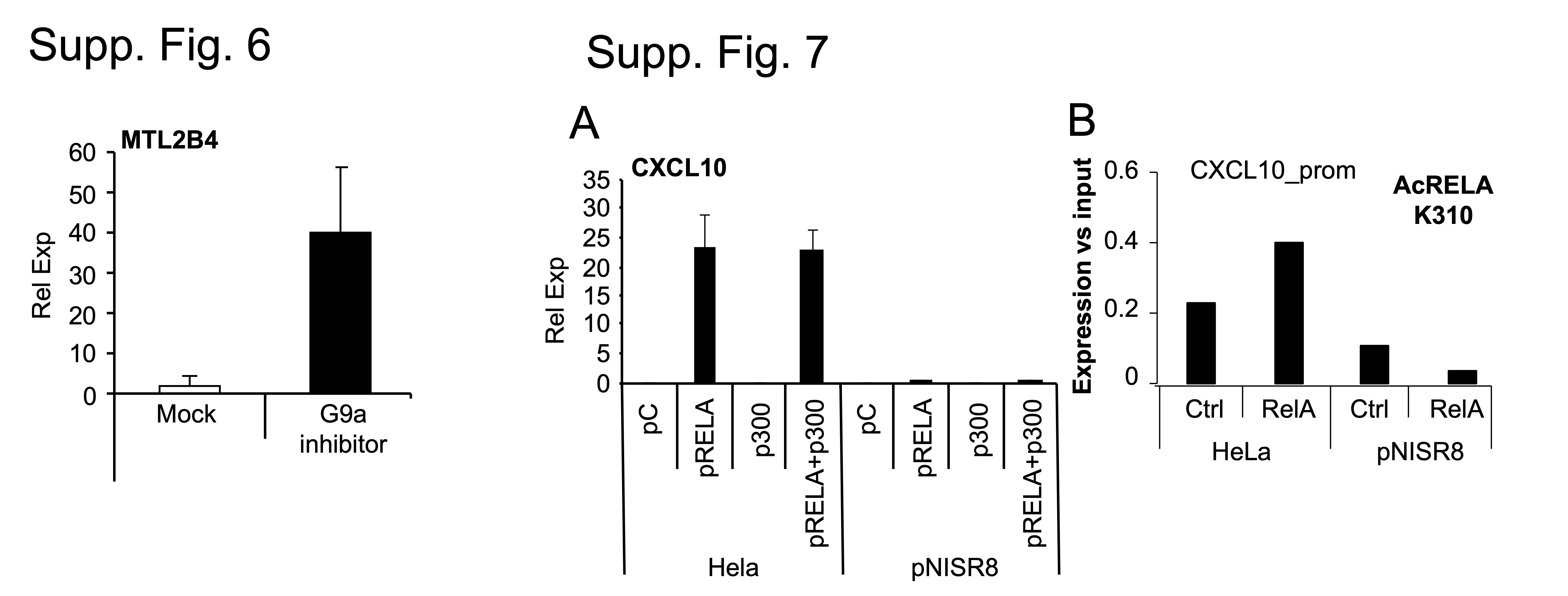

Supplement: Supplementary Figure 6 — Analysis of the functionality of G9a inhibitors. HeLa cells were mock-treated or treated with G9a inhibitors and ERV MTL2B4 RNA expression was evaluated by qRT-PCR. GAPDH mRNA was also evaluated and used as a reference. Error bars indicate standard deviations. [file Image_5.jpg]

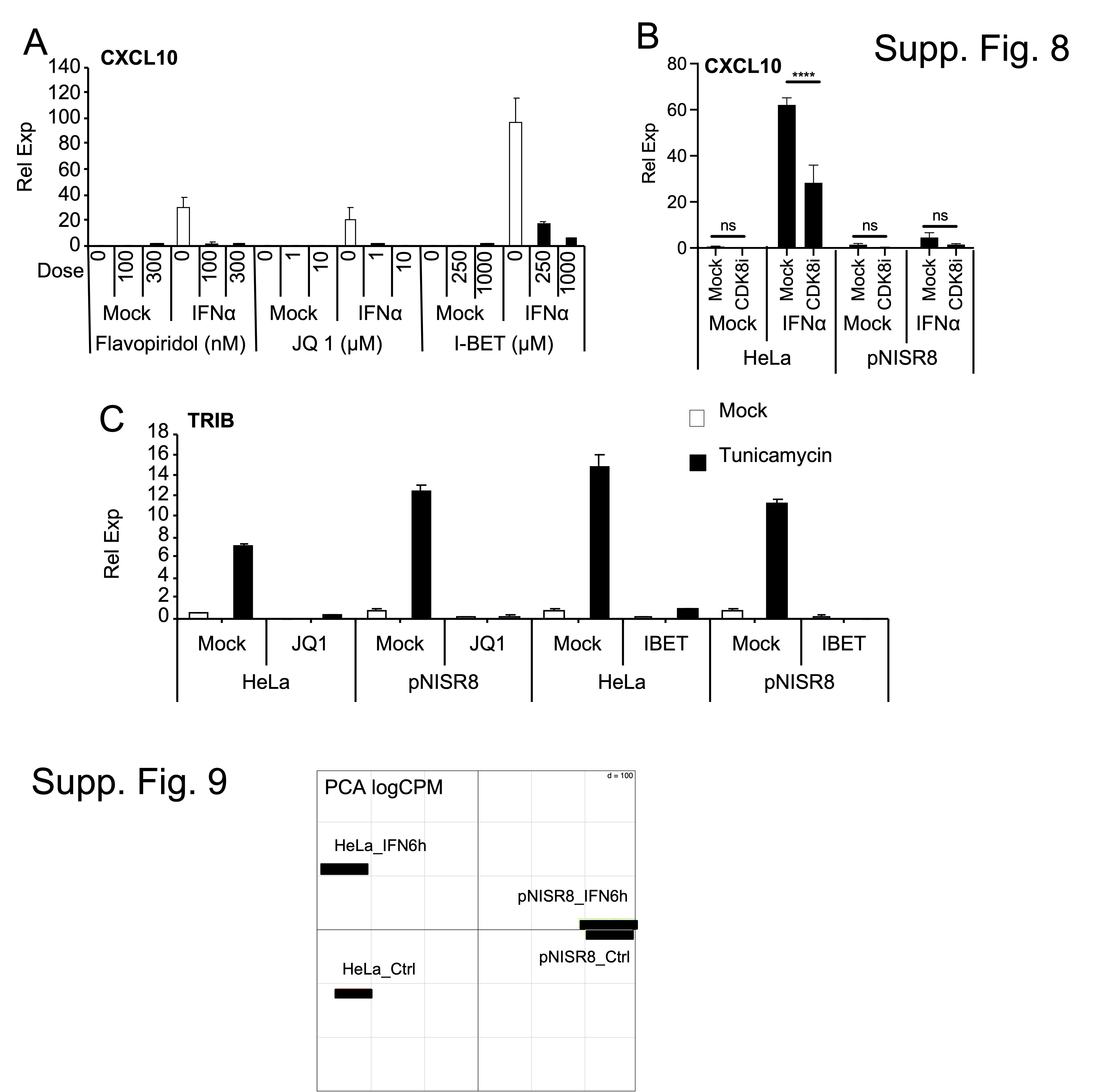

Supplement: Supplementary Figure 8 — Analysis of enhancer dependence of CXCL10 activation and UPR response. (A). CXCL10 mRNA levels in HeLa cells mock-treated or treated with the indicated doses of flavopiridol JQ1, I-BET1 and 0 or 1000U/ml of IFNα for 6h. (B). Similar to A but HeLa and pNISR8 cells were evaluated after treatment with the CDK8 inhibitor CCT251545. (C). TRIB3 mRNA levels of the indicated control cells or cells treated with JQ1 and I-BET1 inhibitors with or without tunicamycin. GAPDH mRNA was also evaluated and used as a reference. Error bars indicate standard deviations. Experiments were performed at least twice and a representative figure is shown. [file Image_6.jpg]

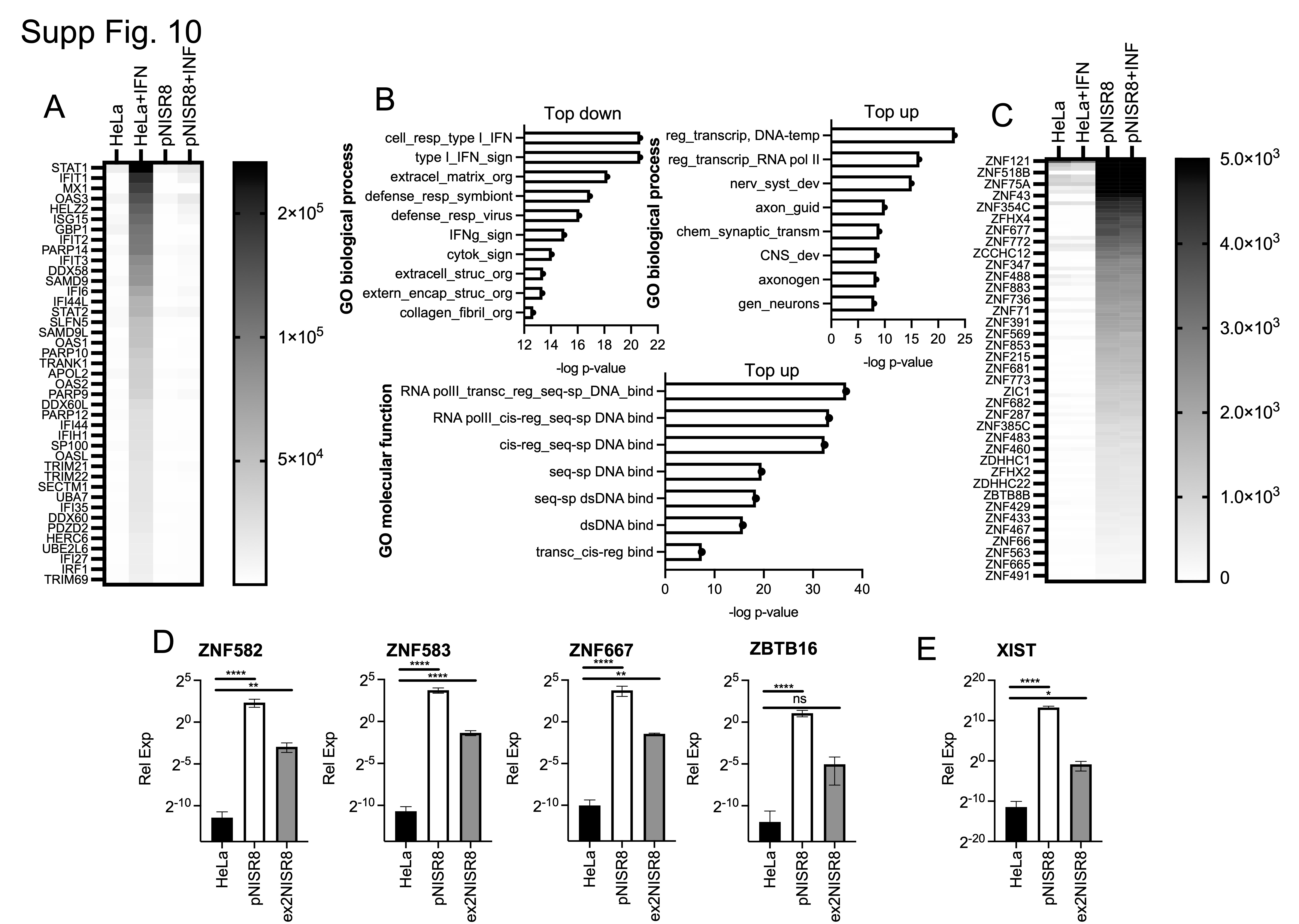

Supplement: Supplementary Figure 10 — Transcriptome analysis of HeLa and pNISR8 cells. (A). Expression levels in the indicated cells of ISGs upregulated over seven-fold in HeLa cells after IFNα treatment. (B). Gene ontology enrichment of biological processes and molecular functions of genes downregulated or upregulated (FC>5) in pNISR8 cells versus IFNα-treated HeLa cells. (C). mRNA levels of Zinc finger proteins upregulated (FC>5) in pNISR8 cells versus HeLa cells in the indicated cells. Only 1/3 of the names are indicated to the left for clarity. (D, E). RNA was isolated from HeLa, pNISR8 and ex2NISR8 cells and the levels of the indicated ZNF transcripts (D) or XIST (E) were evaluated by qRT-PCR. GAPDH mRNA was also evaluated and used as a reference. Experiments were performed three times and a representative image is shown. Statistical analysis is indicated. [file Image_7.jpeg]
